# An Electroactive Filter with Tunable Porosity Based on Glycolated Polythiophene

**DOI:** 10.1002/smsc.202100113

**Published:** 2022-01-28

**Authors:** Johannes Gladisch, Vasileios K. Oikonomou, Maximilian Moser, Sophie Griggs, Iain McCulloch, Magnus Berggren, Eleni Stavrinidou

**Affiliations:** ^1^ Laboratory of Organic Electronics Department of Science and Technology Linköping University SE-60174 Norrköping Sweden; ^2^ Wallenberg Wood Science Center Linköping University SE‐60174 Norrköping Sweden; ^3^ Department of Chemistry University of Oxford Oxford OX1 3TA UK; ^4^ KAUST Solar Center King Abdullah University of Science and Technology (KAUST) Thuwal 23955–6900 Saudi Arabia

**Keywords:** actuators, conjugated polymers, filters, porosities

## Abstract

The porosity of filters is typically fixed; thus, complex purification processes require application of multiple specialized filters. In contrast, smart filters with controllable and tunable properties enable dynamic separation in a single setup. Herein, an electroactive filter with controllable pore size is demonstrated. The electroactive filter is based on a metal mesh coated with a polythiophene polymer with ethylene glycol sidechains (p(g3T2)) that exhibit unprecedented voltage‐driven volume changes. By optimizing the polymer coating on the mesh, controllable porosity during electrochemical addressing is achieved. The pores reversibly open and close, with a dynamic range of more than 95%, corresponding to over 30 μm change of pores’ widths. Furthermore, the pores’ widths could be defined by applied potential with a 10 μm resolution. From among hundreds of pores from different samples, about 90% of the pores could be closed completely, while only less than 1% are inactive. Finally, the electroactive filter is used to control the flow of a dye, highlighting the potential for flow control and smart filtration applications.

## Graphical abstract

An electroactive filter is presented based on a metal mesh coated with a polythiophene polymer with ethylene glycol sidechains (p(g3T2)) that exhibit unprecedented voltage‐driven volume changes. The filters' pores can reversibly open and close upon electrochemical addressing with a dynamic range of more than 95%, corresponding to over 30 μm change of the pores’ widths.

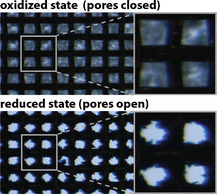

## 1 Introduction

**Figure 1:**
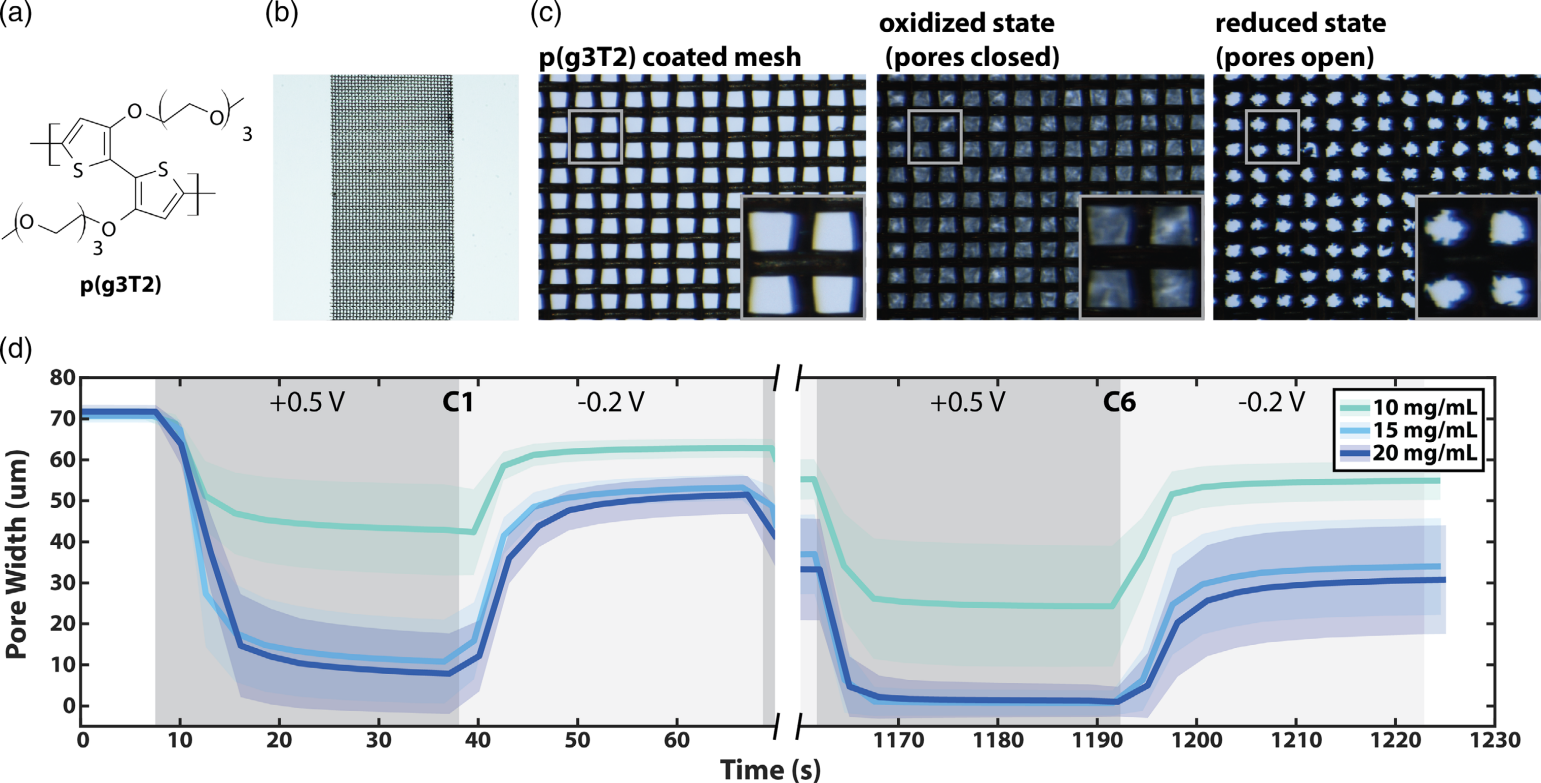
a) Chemical structure of p(g3T2). b) Photograph of a mesh coated with p(g3T2) (15 mg mL^−1^). c) Micrographs of a mesh coated with a 20 mg mL^−1^ solution of p(g3T2) in the pristine, closed (third cycle), and opened (third cycle) state. d) Average ± standard deviation pore widths during electrochemical addressing for meshes coated with polymer solution concentrations of 10, 15, and 20 mg mL^−1^ in the first and sixth cycle (*n* = 3 for each polymer concentration).

Filtering is used in numerous applications such as water treatment,^[^ ^1^ ^]^ food processing,^[^ ^2^, ^3^, ^4^ ^]^ separation of solvents,^[^ ^5^ ^]^ and purification of biopharmaceuticals.^[^ ^6^, ^7^ ^]^ Usually the filter properties, and most importantly porosity, are fixed, requiring the use of different filters for multistep processes. Filters whose properties can be tuned in situ will greatly simplify complex processing and enable new features such as feedback‐regulated filtration. One way to dynamically control the filter properties is to use stimuli−responsive materials whose properties change based on either a physical or chemical stimulus.^[^ ^8^, ^9^ ^]^ Conjugated polymers’ (CPs) properties change when they are electrochemically reduced or oxidized. Specifically, electrochemical modulation of conducting polymers’ volume,^[^ ^10^, ^11^ ^]^ surface charge,^[^ ^12^ ^]^ and wettability^[^ ^5^, ^13^, ^14^, ^15^ ^]^ is attractive for smart filters for controlling porosity or hydrophilicity. Volume changes of CPs have been mainly used in actuators and drug delivery devices,^[^ ^16^, ^17^ ^]^ while their application in smart filters is barely explored.^[^ ^18^, ^19^, ^20^ ^]^ Prönnecke et al.^[^ ^20^ ^]^ demonstrated Polypyrrole‐dodecylbenzenesulfonate (PPy‐DBS)/Al_2_O_3_‐based nanoporous filters where the flow rate of biotin could be decreased by 90% when PPy‐DBS was electrochemically addressed and the porosity of the filter decreased. In another example, Tan et al. demonstrated active PPy‐DBS/PVDF ultrafiltration membranes. By changing the oxidation state of PPy in combination with backwashing, the membrane fouling was alleviated, increasing the flux by 22%. Furthermore, PPy expansion enabled a certain degree of control over humic acid macromolecule filtration. Nevertheless, in these examples, filter porosity control was only achieved in the nanometer range. PPy‐DBS typically exhibits an out‐of‐plane volume change of 40%, limiting the dynamic range of controllable porosity.^[^ ^11^, ^21^ ^]^ In contrast, materials with larger volume change abilities will enable extended dynamic range of pore dimension control. Recently, we reported that polythiophenes functionalized with ethylene glycol side chains exhibit unprecedented reversible volume changes when electrochemically addressed. A polythiophene with triethylene glycol side chains (p(g3T2), **Figure** **1**a, can reversibly change in volume by 250% over hundreds of switching cycles.^[^ ^10^, ^22^, ^23^ ^]^ In addition, the volume change magnitude can be modulated via the applied potential within the ±0.5 V range and via chemical design of the polymer offering a handle for optimization and customization.^[^ ^22^ ^]^

In this work we harness the unprecedented volume change of p(g3T2) to develop smart filters with tunable porosity in the micrometer scale. By coating a metallic mesh with p(g3T2) we convert it to a smart filter whose pores’ dimensions can be controlled by the applied voltage.

## 2 Results and Discussion

To fabricate the electroactive filter, metal meshes with square‐shaped pores of 73.7 × 73.7 (±2.7) μm^2^ were coated with p(g3T2) (see Figure 1a). Initially, we optimized the polymer‐coating procedure to achieve a homogenous and reproducible layer. Dip coating and passive drying in air led to inhomogeneous polymer coating with excessive material in parts of the mesh, while spin coating led to insufficient material on the mesh (see Figure S1a,b, Supporting Information). In contrast, by dipping the mesh in the polymer solution and drying it via mechanical shaking, the coating became homogenous and reproducible, as shown in Figure 1b,c and S1–S3, Supporting Information.

We hypothesized that the polymer layer formation will depend on the polymer solution concentration. Therefore, we coated meshes using polymer solution concentrations of 10, 15, and 20 mg mL^−1^. To ease the discussion that follows, we will name the meshes based on the polymer concentration used for their coating.

We found that the higher the concentration of polymer during coating, the more the material is being deposited on the mesh, especially at the thread joint sections, as determined from scanning electron microscopy images (Figure S2, S3, Supporting Information and **Table** **1**).

**Table 1:** Polymer‐coating properties

Concentration [mg mL^−1^]	Polymer thickness [μm]	% Area covered at joint [%]
10 (*n* = 3)	0.33 ± 0.04	14 ± 3.9
15 (*n* = 3)	0.43 ± 0.09	28.9 ± 2.6
20 (*n* = 3)	0.79 ± 0.10	43.3 ± 10.1

To investigate whether the meshes’ porosity can be controlled electrochemically, the meshes were mounted on a planar three‐electrode electrochemical setup and time‐lapse microscopy was performed. From the acquired micrographs, the pore widths were computationally extracted based on gray values, as described in detail in the Experimental Section.

Figure 1c,d shows that the pores of the mesh can be controlled depending on the oxidation state of p(g3T2) (the whole switching sequence can be found in Figure S4, Supporting Information). The pores’ width becomes smaller when p(g3T2) is oxidized (+0.5 V) and subsequently increases when the polymer is reduced (−0.2 V) within a few seconds of voltage application (Figure S5, Supporting Information). When p(g3T2) is oxidized, the volume increases because of inclusion of anions and water from the electrolyte. The anions serve as the dopant moieties that compensate for the injected positive electronic charges on the polymer backbone. During reduction, on the other hand, the polymer volume decreases as the anions with accompanied water molecules are being expelled to preserve electroneutrality.^[^ ^10^ ^]^

During the first electrochemical addressing, the pores of 15 and 20 mg mL^−1^ meshes in the oxidized state closed by −85% (to 10.8 ± 10.2 μm) and by −89% (to 7.9 ± 9.8 μm), respectively. In contrast the pores of 10 mg mL^−1^ meshes closed only by −40% (to 42.9 ± 11.1 μm). When the meshes were electrochemically reduced, the pores opened but they did not recover fully to their initial open state (see also Figure S6a,b, Supporting Information). In 10, 15, and 20 mg mL^−1^ meshes, pores recovered to −12% (62.9 ± 2.3 μm), −25% (53.3 ± 3.1 μm), and −28% (51.5 ± 4.6 μm) of their original values, respectively. With increasing cycle number (switching between +0.5 and −0.2 V), the pores closed almost completely, reaching 0.7 ± 3 μm (−99%) and 1 ± 3.6 μm (−99%) average width in 15 and 20 mg mL^−1^ meshes for the sixth switching cycle. In contrast, for the 10 mg mL^−1^ meshes, the pores closed only up to 24.3 ± 14.8 μm (−66%) width. However, the width in the open state was also smaller, reaching 54.8 ± 4.7 (−23%), 33.8 ± 11.8 (−52%), and 30.6 ± 13.2 μm (−57%) for 10, 15, and 20 mg mL^−1^ meshes, respectively.

The trends can be understood based on p(g3T2) volume change during electrochemical cycling. p(g3T2), as other CPs, exhibits an irreversible volume change during the first cycle that is a result of molecular rearrangements and water retention during electrochemical switching.^[^ ^10^ ^]^ As the volume change magnitude depends on the initial polymer volume, from one side, we can achieve larger volume change, but on the other side, we observed larger irreversibility for meshes coated with 15 and 20 mg mL^−1^. Furthermore, p(g3T2)'s absolute volume and irreversible change increase over cycling, resulting in smaller pore widths both in the closed and in the open state.^[^ ^22^ ^]^ However, the dynamic range that can be achieved for 15 and 20 mg mL^−1^ meshes is high, with more than 96% change on pores widths between open and closed states corresponding to a change of pores width of 36.2 ± 10.3 and 32.2 ± 12.1 μm, respectively, for 15 and 20 mg mL^−1^ (Figure S6c,d, Supporting Information).

**Figure 2:**
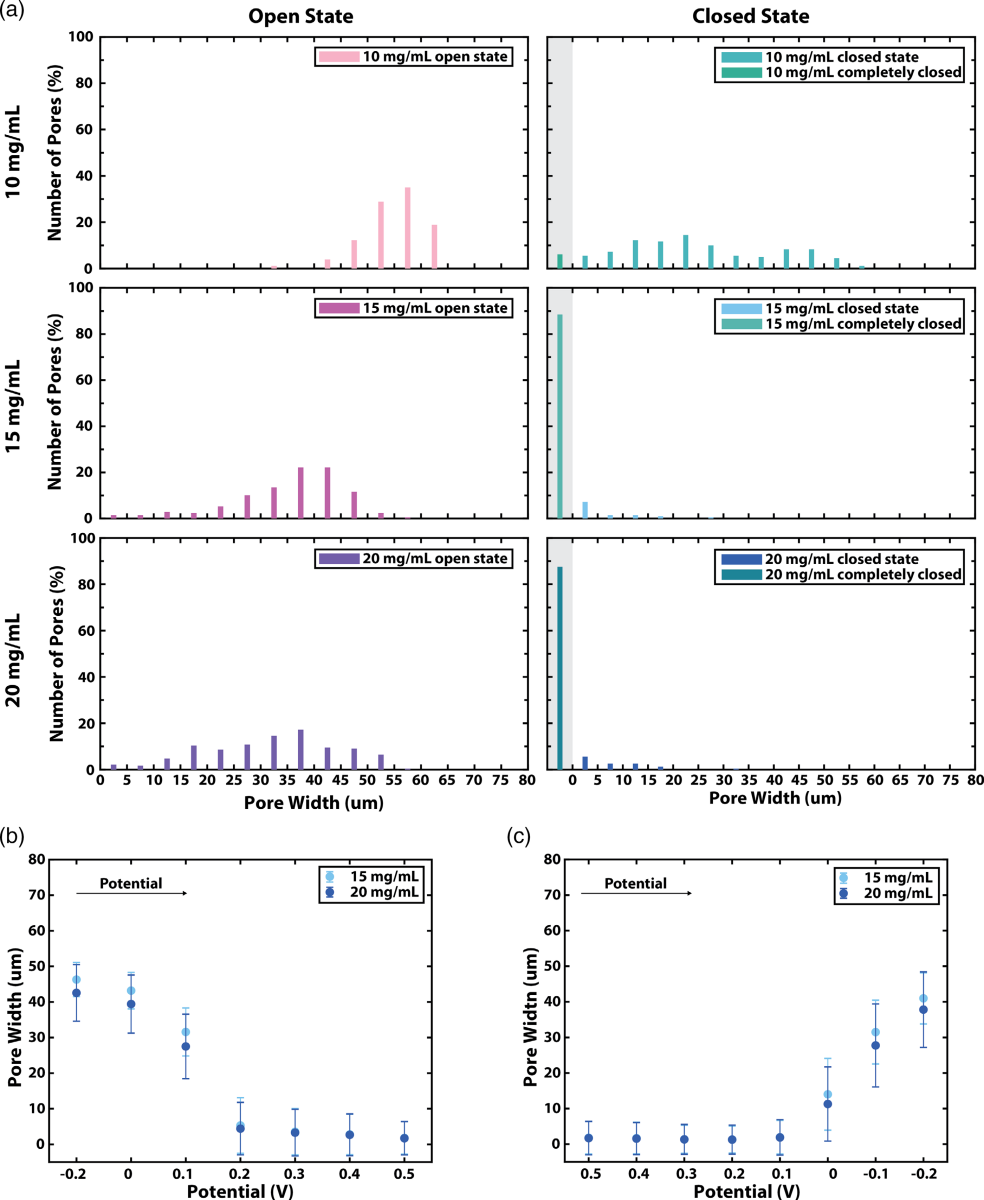
a) Distribution of the pore widths of meshes coated with different polymer concentrations, in closed (shades of turquoise−blue) and reopened (shades of purple−pink) state it the sixth cycle (bin size: 5 μm). The bars with the gray background in the closed state correspond to the pores that have a width of 0 μm, that is, are fully closed. Pore widths at different potentials in 0.1 V steps during b) closing and c) opening (all performed in 0.01 m KCl in a three‐electrode setup).

To gain more insight into the variation across individual pores at different coating concentrations, we assessed the distribution of the pores’ widths (**Figure** **2**a and S7, Supporting Information, grouped in 5 μm‐width range categories (bins)). For concentrations of 15 and 20 mg mL^−1^ in the closed state (oxidized polymer), ≥87% of pores close completely (no pixels in the pore area are above the gray threshold), while ≥93% of all pores were between 0 μm < *d* < 5 μm in the sixth cycle. In the same state for the 10 mg mL^−1^ mesh, we observed a much larger variation with only 6% of the pores closing completely and only ≈12% of the pores below 5 μm. For the open state, 79% of the pores of 15 mg mL^−1^ meshes were between 25 and 50 μm. For the 20 mg mL^−1^ meshes, the variation was larger, with 79% of the pores having a width between 15 and 50 μm. In contrast for the 10 mg mL^−1^ mesh, we observed a smaller variation in the open state as 95% of the pores was between 45 and 65 μm. Therefore, there is a tradeoff between control of the closed versus the open state. As discussed previously, this is a result of the increasing irreversible volume change and the absolute volume of p(g3T2) with cycling. Finally, we determined the number of inactive pores. We defined pores as inactive if their width change during opening or closing is smaller than 5% of the average change for the respective cycle. We found zero inactive pores for all meshes during the first cycle. At later cycles, less than 1% of the pores were inactive for 15 and 20 mg mL^−1^ during closing and 4% for the 20 mg mL^−1^ during opening.

Overall, the assessment shows that the 10 mg mL^−1^ mesh is the worst performing in terms of dynamic range and variability of the pores’ control. This can be attributed to the nonsufficient coating of the mesh with lower polymer concentration.

Next, we investigated whether we could control the pore size of 15 and 20 mg mL^−1^ meshes through voltage with a higher resolution achieving intermediate states between the fully open and fully closed state by varying the applied voltage with 0.1 V steps (Figure 2b,c). When the pores are open at *V* = −0.2 V, their closing can be controlled gradually within 0 and +0.2 V. For voltages higher than +0.2 V, no further changes are observed. When the pores are fully closed at *V* = +0.5 V, their opening can be controlled in the voltage range between +0.1 and −0.2 V. By applying 0.1 V voltage steps, we could reach resolution on the pore width of about 10 μm. However, in principle, by applying smaller voltage steps we can increase the resolution of pore size control. We observed that the degree of opening of the pore depends not only on the applied voltage magnitude but also on the history of addressing. The memory effect observed in CPs is a result of the changes in the polymer matrix due to exchange of ions and water with the electrolyte during electrochemical addressing.^[^ ^24^, ^25^ ^]^

We then investigated the performance of the electroactive meshes over extended cycling. A mesh coated with a polymer concentration of 15 mg mL^−1^ was switched between open and closed states for 20 cycles (Figure S8, Supporting Information). We observed similar trends as in the detailed investigation described for the first electrochemical cycles; the pores’ width, both in open and in closed state, decreased with cycling. The closed state stabilized after 7 electrochemical cycles, while the open state stabilized after 13 cycles (Figure S8a, Supporting Information). In the 20th cycle, the pore widths were 43 ± 8.8 μm in the open and 10.9 ± 10.6 μm in closed state corresponding to a change in pore width of 32 μm.

We also investigated if the polymer‐coated mesh can be reused when dried in contracted state after electrochemical cycling. The polymer volume after drying decreased and became comparable with the pristine state (Figure S9, Supporting Information), demonstrating that the volume change during electrochemical addressing is mostly due to retention of water. The dried mesh could be electrochemically readdressed, resulting in expansion and contraction of the polymer volume. However, the pores did not close as significantly as before. This can be explained by a loss of active material most probably during the removal of the electrolyte around the mesh during the drying process.

**Figure 3:**
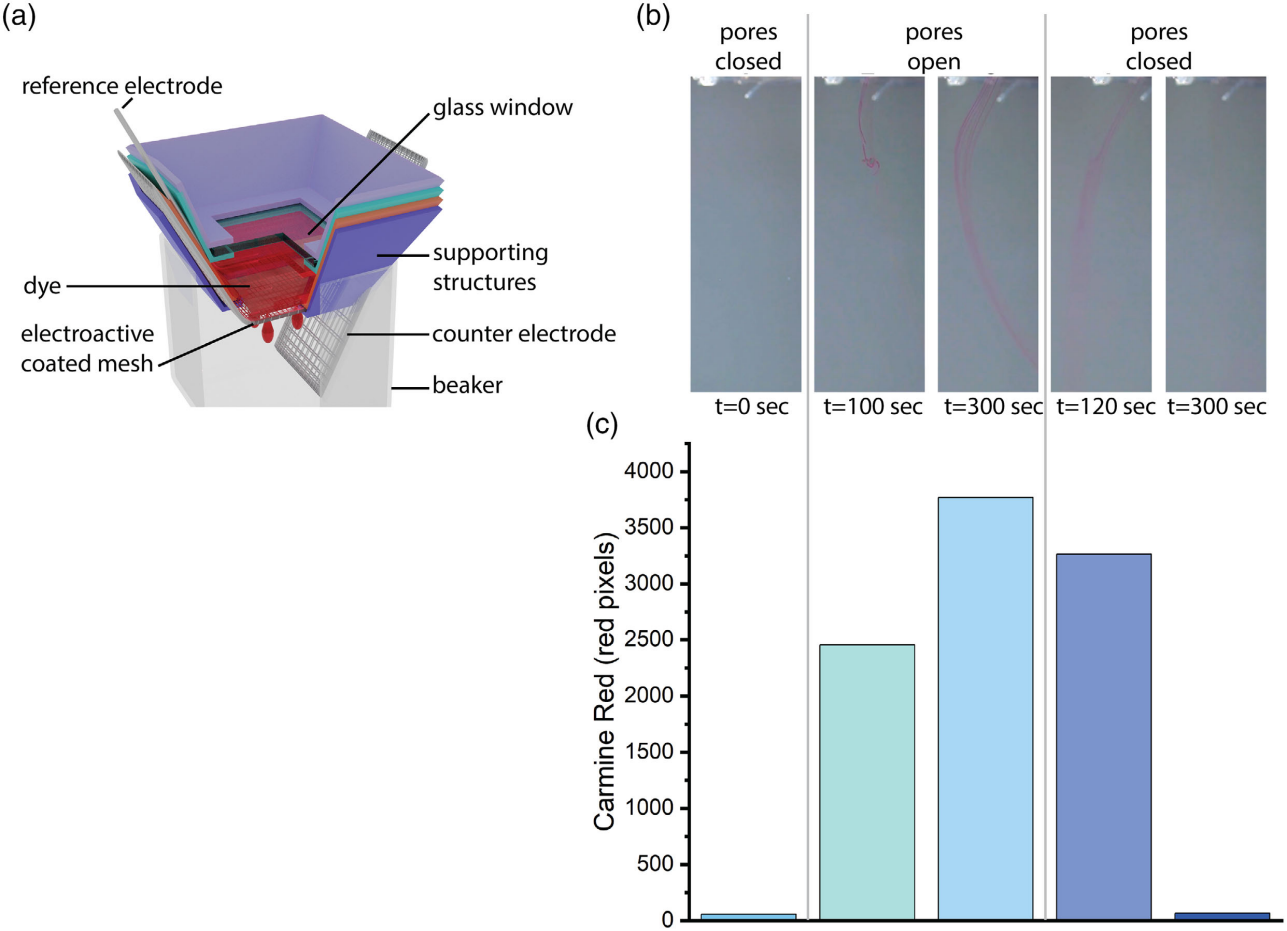
a) Schematic of the electroactive filter setup. b) Dye flow from the reservoir into the bulk electrolyte with the electroactive filter in different states and timepoints. c) Number of pixels in the red color range depicting the presence of red dye in the different states of the electroactive filter.

As a proof of concept of a smart filter application, we constructed an electroactive filter for controlling the flow of a dye. We chose meshes coated with 15 mg mL^−1^ polymer solution as they performed best as described in detail in the previous sections. The meshes were mounted in a custom 3D‐printed setup (depicted in **Figure** **3**a). First the pores of the filter were closed by applying +0.5 V (polymer in expanded state) and then 200 μL commercial carmine red‐based food coloring was added in the chamber on top of the filter. No leakage was observed, demonstrating that the filter pores could be closed to a degree that a molecular dye could not pass. Then, the filter pores were opened by applying −0.2 V, thereby contracting the polymer. The pore opening led to the release of the dye (Figure 3b,c) with a delay of ≈100 s from the onset of the voltage. We speculate that the delay is a result of the time required for the dye to pass from the 3D‐printed setup and be visible to the camera. When we closed the pores again (second cycle), the trace of the dye started to fade, and the release stopped after ≈120 s. An identical release profile was observed for the third cycle as well. The experiment was repeated for three electroactive filters, all showing consistent results, not just in terms of general flow control but also in terms of the kinetics of the release and flow control (Figure S10, Supporting Information).

## 3 Conclusion

In this work, we demonstrated an electroactive filter with tunable porosity in the micrometer range. By simply coating meshes with the conducting polymer p(g3T2), we could control the pores’ widths electrochemically. By optimizing the polymer coating, we achieved a dynamic range between 0 and 32 μm on the pores’ width, demonstrating the first tunable mesh in the micrometer scale. 87% of the pores could be closed completely, while less than 1% of the pores were inactive. Furthermore, the pores’ width could be controlled via the applied voltage by 10 μm steps. As a proof of concept, the smart filter was used to effectively control the flow of a dye molecule. The p(g3T2)‐based filters have high control on the closed state, while the open state requires further optimization as there is variability on the pores width (79% between 25 and 50 μm), which is a result of irreversible volume change and the increase in the absolute polymer volume during electrochemical cycling. The filter is therefore not optimized for high‐resolution sorting of microsubstances, but it can be used for sorting with a cutoff dimension. Furthermore, the simple but effective way to control porosity can have implications not only in filtering and separation methods but also in microfluidics and drug delivery. Better control of the open state can be achieved by further optimizing the polymer coating or using a polymer that exhibits high volume change but less irreversibility. Even though in this work we used mesh sizes with areas in the cm^2^ range, the solution‐based processing enables large‐scale applications.

## 4 Experimental Section

4.1

4.1.1

##### Polymer Synthesis

p(g3T2) was synthesized as described previously.^[^ ^10^ ^]^

##### Electroactive Mesh Fabrication

Polymer solutions of different concentrations were prepared using chloroform as the solvent. The solutions were gently shaken until the polymer was dissolved.

The meshes (screen printing meshes 80 μm pore size, Coated Screens Scandinavia AB) were dipped into the polymer solutions in a vial and immediately transferred into a vial placed on the vortex mixer (Scientific Industries Vortex Genie 2, maximum intensity) and shaken thoroughly until dried.

##### Electroactive Mesh Characterization

For the coating optimization experiments, the meshes were mounted in a custom setup with a poly(dimethylsiloxane) (PDMS) well and a glass lid to prevent evaporation and improve the visibility. The pore size variation following electrochemical stimulation was then monitored with a microscope (Nikon SMZ1500 with Nikon WD 45 lens and a Nikon DS‐Fi1 camera controlled with Nikon NIS Elements software 3.22.15) in a three‐electrode arrangement with the coated meshes as working electrode, a Pt wire as a counter electrode, and Ag/AgCl wire as pseudoreference. 0.01 m KCl served as the electrolyte in all experiments (Metrohm μAutolab Type III potentiostat with NOVA 2.1 software).

The pore sizes in the microscope images were processed with a MATLAB (2018 a) script based on gray thresholds (timestamps were extracted from the .nd2 files with the Bio‐Formats plugin^[^ ^26^ ^]^). More precisely, based on the pictures in the pristine state, the region of interest of the individual pores was defined (the process is depicted in Figure S11, Supporting Information). Pores containing bubbles were manually excluded. Nonetheless, for each sample, at least 49 pores were considered. The exact number of pores is specified in Table S1, Supporting Information. Having the individual pores defined, the opening/closing state of the individual pores in all the acquired images were determined based on the gray threshold. Here, pixels brighter than the threshold were considered as open and those below as closed. The number of pixels were then summed up and the width of the area covered by the sum of pixels was calculated (Figure S11d, Supporting Information).

For every dataset, a set of pores was automatically selected randomly to serve as control pores for a representative open and closed state, where the pore widths were manually acquired by ImageJ as well and compared with the computationally acquired values (Figure S12, Supporting Information). The pore dimensions were manually determined by the average of four values 45° apart for the respective features.

The datasets of similarly prepared meshes were pooled according to Equation S1, Supporting Information, to acquire the mean and standard deviations of the meshes coated with the respective polymer concentration.

The images to calculate the polymer thickness and material deposition were obtained by SEM microscopy (Zeiss‐Sigma 500 Gemini). The coating thickness was measured with the use of ImageJ software, and the length of the coating was extracted by SEM images similar to Figure S3e, Supporting Information, where the difference between the mesh and the polymer can be observed. The polymer appeared darker than the metallic mesh. To retrieve the cross‐sectional images, the mesh was cut and a slight curvature in the edge of the cross section was observed. In total, three meshes of 10, 15, and 20 mg mL^−1^ concentrations were characterized and the polymer thickness and the area coated were measured based on six points in three different joints for each case.

##### Flow Control Measurements

For the flow control experiments, p(g3T2)‐coated metal meshes, with limited active area to about 5 × 5 mm by a parafilm, were placed in a custom 3D‐printed setup in a beaker filled with 0.01 m KCl (Figure 3a). The custom 3D‐printed setup comprised a chamber above of the mesh to hold the dye and a glass slide on top of the chamber, allowing the observation of the mesh with the microscope. At the same time, a second camera was placed in front of the setup to monitor the dye release. Initially 5 switching cycles (+0.5–−0.2 V) of 1 min/30 s each were performed to reduce the impact of break‐in effects on the experiment. The controlled release was subsequently observed for three cycles of 5 min each. After about 100 s, in the first expansion phase, the dye (Dr. Oetker Red Food Dye, Carmine Red, sugar, water) was gently placed in the chamber on top of the mesh. Then, the flow of the dye following the pore size modulation was observed with a camera in front of the beaker.

##### Image Processing

For the photo extraction, white color balance was applied with Adobe Premiere Pro to the time‐lapse video recorded with the camera (Sony A7iii, Macro Lens Sony SEL‐90M28G) to accurately match the colors of the setup.

ImageJ was used for the red pixel calculation, and with the use of Color Counter plugin, the colors of every pixel were extracted and then categorized to the different color ranges. The pixels of the red color range were taken into account to quantify the presence of the red dye in each cycle step.

## Conflict of Interest

The authors declare no conflict of interest.

## Author Contributions

E.S. and M.B. conceived the project. J.G. and V.K.O. performed the experiments and analyzed all data. M.M., S.G. and I.M. synthesized materials. J.G., V.K.O. and E.S wrote the paper with input from all authors. E.S. supervised the work.

## Supporting information

Supplementary Material

## Data Availability

Research data are not shared.
