# Supplementary material for: An Electroactive Filter with Tunable Porosity Based on Glycolated Polythiophene
Source: Small Sci. 2022 Jan 28;2(4):2100113. doi: 10.1002/smsc.202100113 (PMC11935962; doi:10.1002/smsc.202100113)
Supplement: Supplementary file 1 — Supplementary Material [file SMSC-2-2100113-s001.pdf]

Supporting Information

An electroactive filter with tuneable porosity based on glycolated polythiophene

*Johannes Gladisch, Vasileios K. Oikonomou, Maximilian Moser, Sophie Griggs, Iain McCulloch, Magnus Berggren, Eleni Stavrinidou**

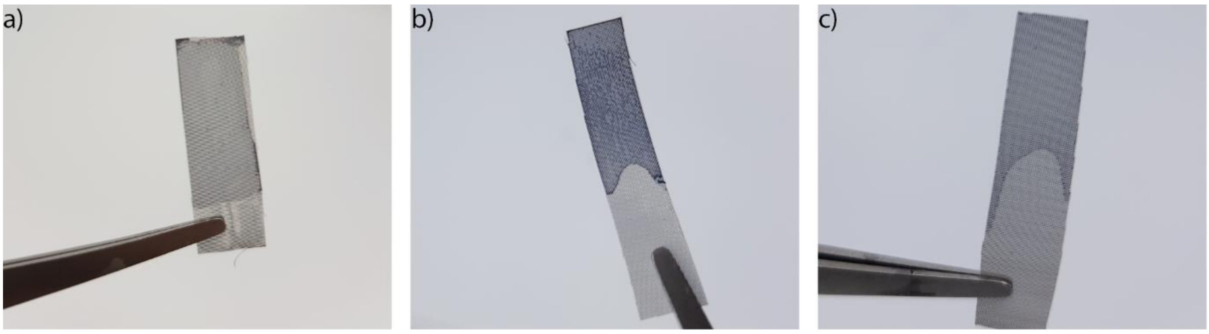

Figure S 1: Mesh coating with 20 mg/mL polymer solution via a) spin coating, b) dip-coating (drying in air) and c) dip-coating (drying with mechanical shaking).

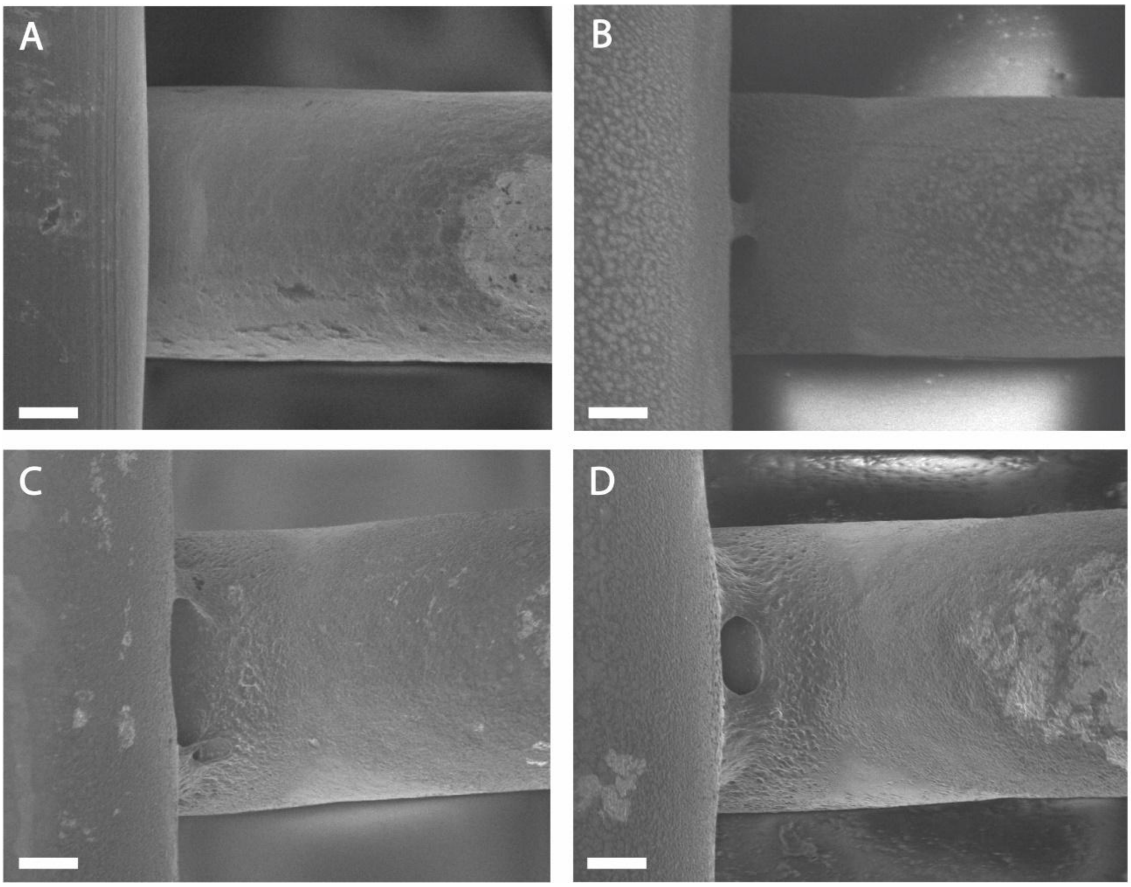

Figure S 2: SEM images of a) uncoated mesh and dip coated meshes with mechanical shaking drying for polymer solution concentration of b) 10 mg/mL, c) 15 mg/mL and d) 20 mg/mL (scalebar: 10 μm)

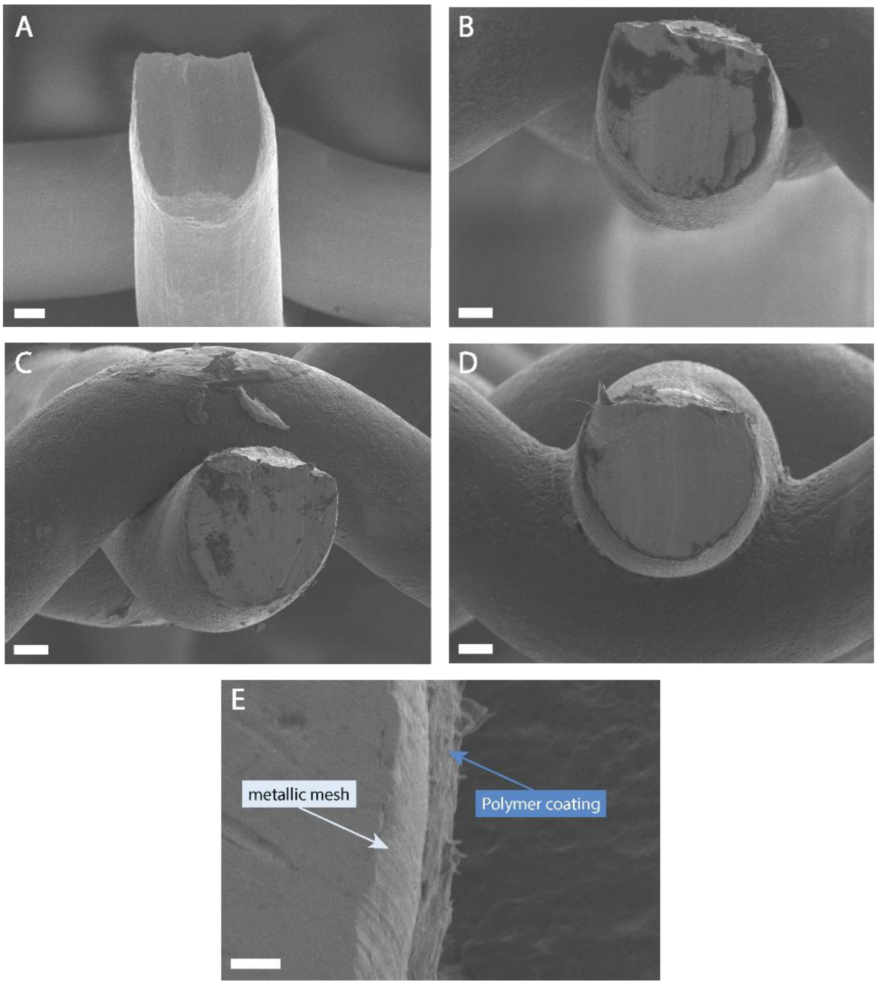

Figure S 3: SEM images of meshes cross sections that are dip coated with mechanical shaking drying for polymer solution concentration of a) uncoated b) 10 mg/mL, c) 15 mg/mL and d) 20 mg/mL (scalebar: 10 µm) e) zoomed image of the cross section (15mg/mL) that shows clearly the difference between the mesh and the polymer coating (scalebar: 2µm)

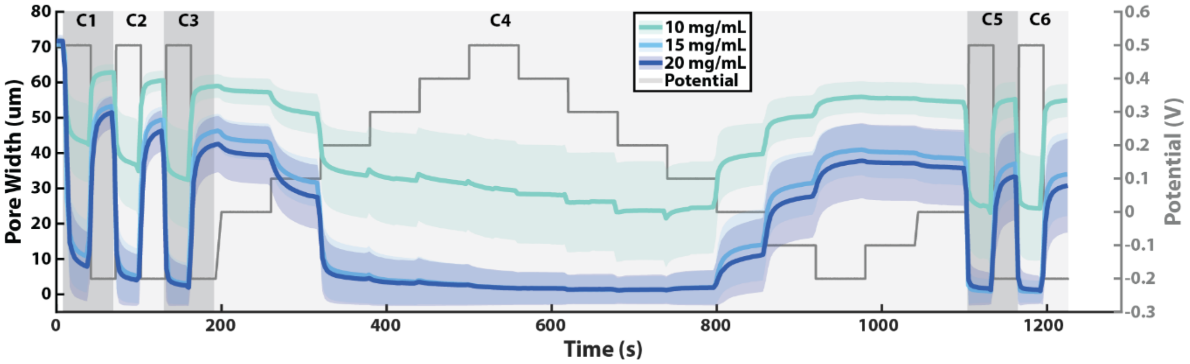

Figure S 4: Overview of the average pore widths during the electrochemical switching sequence for different polymer coating concentrations of 10, 15 & 20 mg/mL

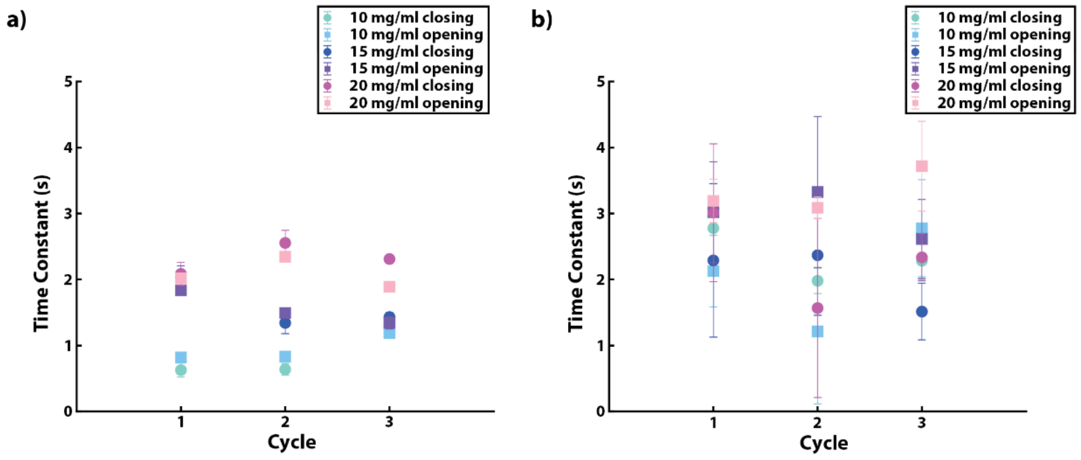

Figure S 5: Pores opening and closing time constants, extracted from a) the current response and b) the microscopy images

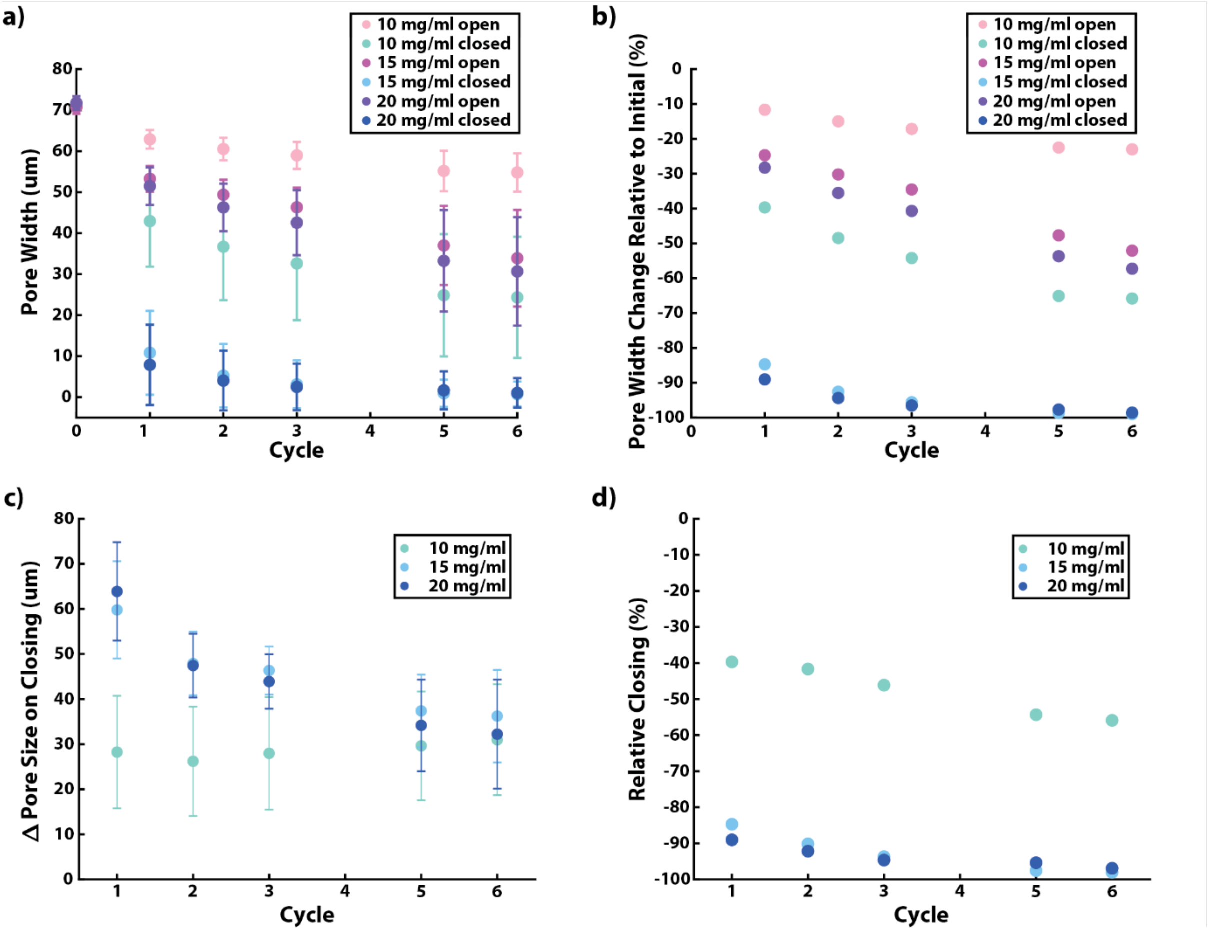

Figure S 6: Pores average characteristics at different electrochemical cycles. a) Average pore widths in open and closed state b) Corresponding relative change of the pore widths in open and closed state with respect to initial pore width. c) Average pore widths change between open and closed state in µm and d) corresponding values in percent.

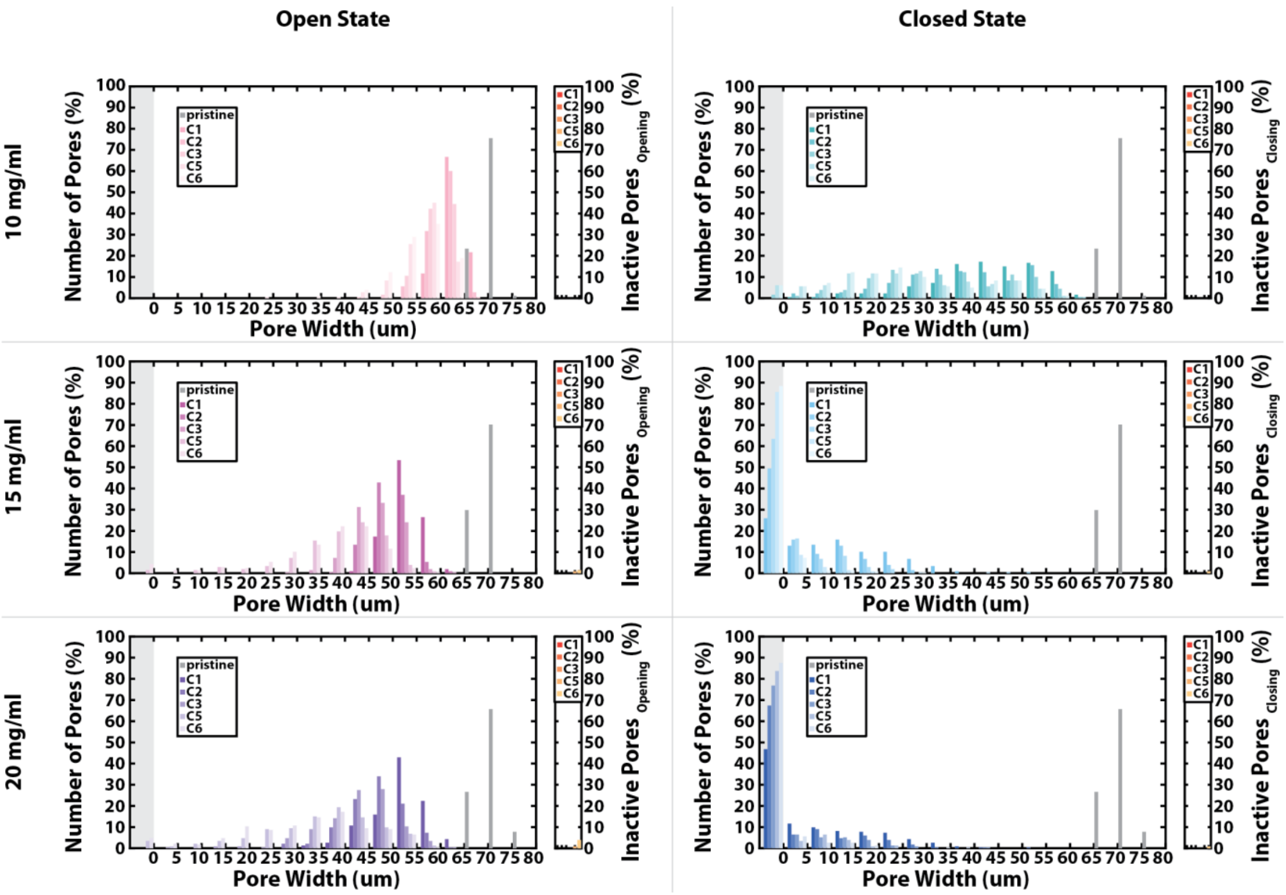

Figure S 7: Bar charts of the distributions of the pore sizes in the opened and closed state for the different concentrations at different cycles (C1-6)

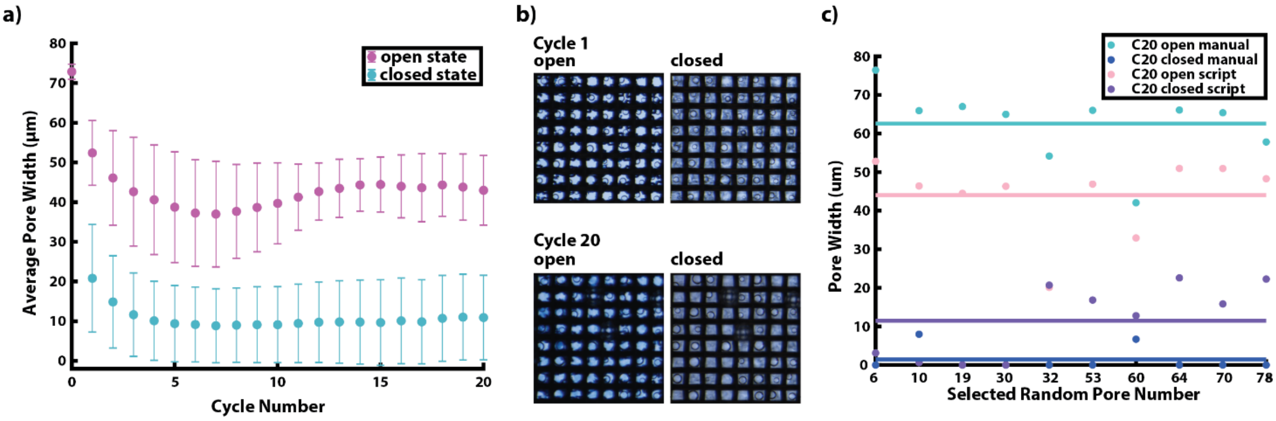

[redacted] Results of the proof-of-concept study of 20 switching cycles of a mesh coated with 15 mg/ml, with a) average widths of the pores and b) images of the 1st and 20th cycle c) comparison between computationally and manually assessed pore widths in the 20th cycle.

In Figure S8 c, a comparison between computationally and manually processed, randomly selected, pores in the 20th cycle are presented. It is to note that in the case of the 20th cycle the script on the one hand underestimates the open state, while on the other hand it overestimates the pore widths in the closed state compared to the manual processing. Consequently, the actual dynamic range of the mesh in the 20th cycle might be underestimated as well. (Similar investigations were also performed for the comparative investigations of the coating at different concentrations between for up to 6 cycles (see also Figure S12)). In the present case,

the offset between the manually and computationally acquired pore dimensions can be explained by a lack of contrast between the polymer in the expanded state and the background in contracted state, making it hard to set an appropriate grey value threshold (see Figure S8 b).

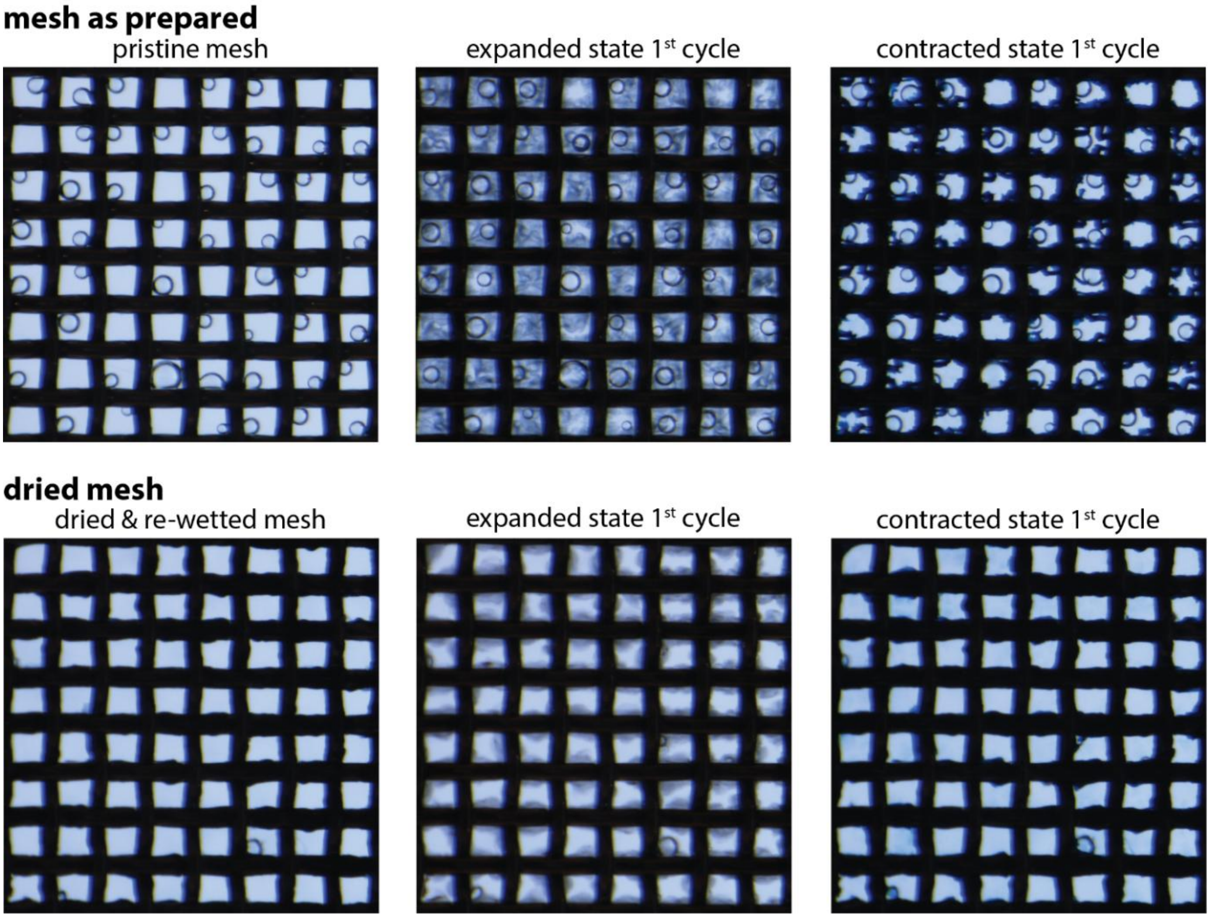

Figure S 9 Micrographs of a mesh during a switching experiment (upper row) and a follow up switching experiment after the same mesh has been dried (lower row).

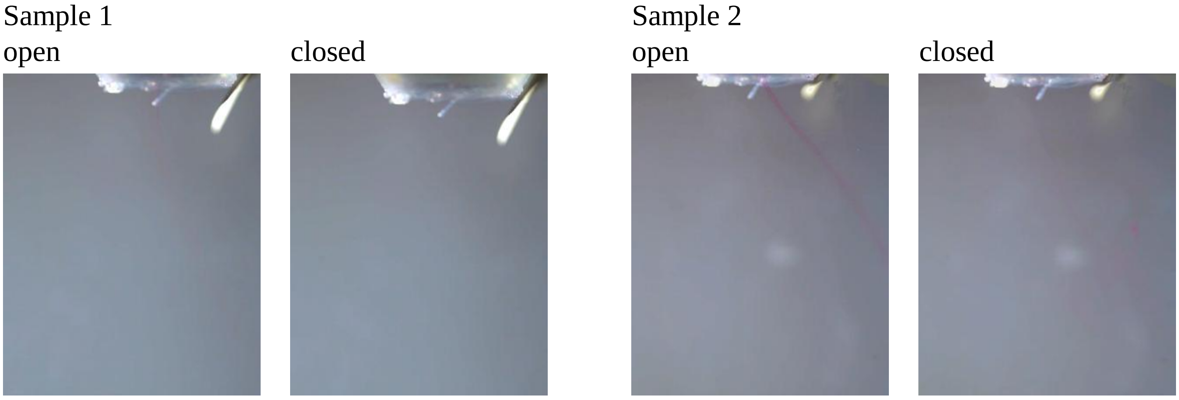

Figure S 10: Dye flow through the electroactive filter in two different samples

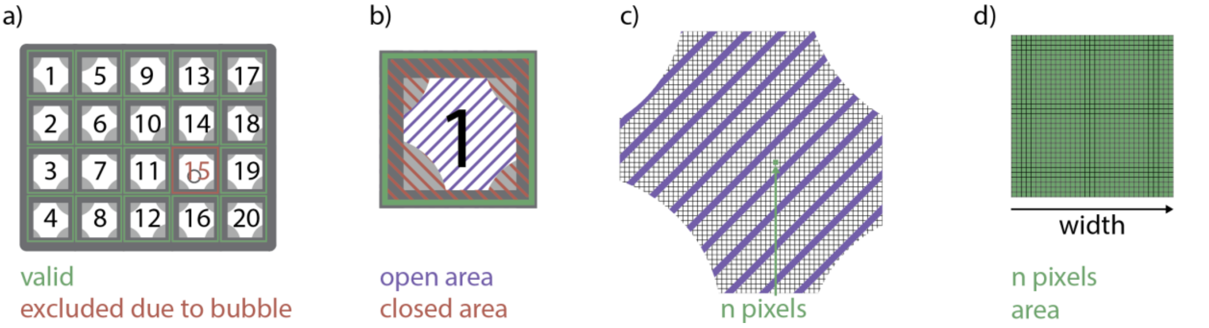

Figure S 11: Schematic representation of the image processing procedure to extract the pore widths of the meshes

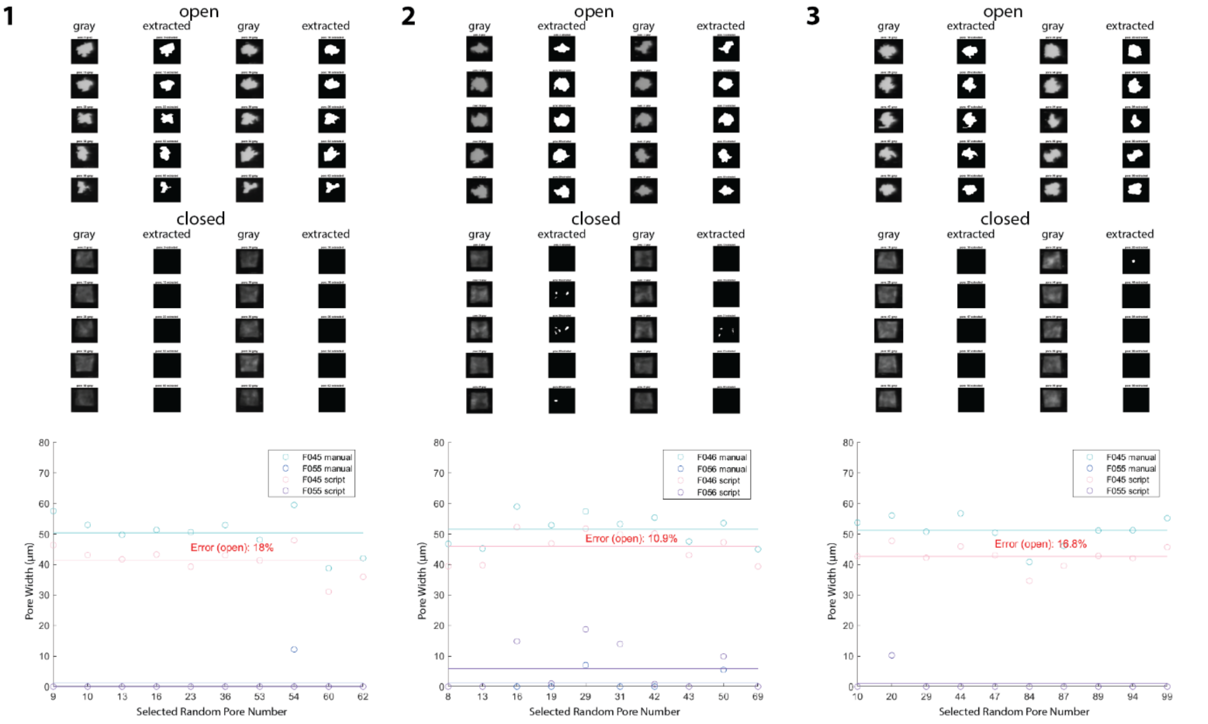

Figure S 12: Examples of the computationally processed randomly selected pores in open state (on top) and closed state (middle) as well as the difference between manually and computationally acquired pore widths of the respective randomly selected pores (bottom). For 3 different samples prepared with 20 mg/mL polymer concentration.

Table S 1: Number of pores considered in the samples for the different coating polymer concentrations

Sample number	10 mg/mL	15 mg/mL	20 mg/mL
**1**	57	57	63
**2**	74	84	107
**3**	49	67	63

Pooled mean:

$$\bar{X}_{pooled} = \frac{\sum(\bar{X}_n \cdot k_n)}{\sum k_n}$$

Pooled standard deviation:

$$SD_{pooled} = \sqrt{\frac{\sum((k_n - 1) \cdot SD_n^2)}{\sum k_n - n}}$$

Equation S 1: equations applied to calculate the pooled mean and standard deviations (k=sample size)
